# Engineering *Corynebacterium crenatum* to produce higher alcohols for biofuel using hydrolysates of duckweed (*Landoltia punctata*) as feedstock

**DOI:** 10.1186/s12934-015-0199-3

**Published:** 2015-02-07

**Authors:** Haifeng Su, Juan Jiang, Qiuli Lu, Zhao Zhao, Tian Xie, Hai Zhao, Maolin Wang

**Affiliations:** Key Laboratory of Bio-resources and Eco-environment of the Ministry of Education, College of Life Sciences, Sichuan University, Chengdu, 610064 Sichuan PR China; Bioenergy Laboratory, Chengdu Institute of Biology, Chinese Academy of Sciences, Chengdu, 610041 Sichuan PR China

**Keywords:** *Corynebacterium crenatum*, Bioengineering, Duckweed, Higher alcohols

## Abstract

Early trials have demonstrated great potential for the use of duckweed (family *Lemnaceae*) as the next generation of energy plants for the production of biofuels. Achieving this technological advance demands research to develop novel bioengineering microorganisms that can ferment duckweed feedstock to produce higher alcohols. In this study, we used relevant genes to transfer five metabolic pathways of isoleucine, leucine and valine from the yeast *Saccharomyces cerevisiae* into the bioengineered microorganism *Corynebacterium crenatum*. Experimental results showed that the bioengineered strain was able to produce 1026.61 mg/L of 2-methyl-1-butanol by fermenting glucose, compared to 981.79 mg/L from the acid hydrolysates of duckweed. The highest isobutanol yields achieved were 1264.63 mg/L from glucose and 1154.83 mg/L from duckweed, and the corresponding highest yields of 3-methyl-1-butanol were 748.35 and 684.79 mg/L. Our findings demonstrate the feasibility of using bioengineered *C. crenatum* as a platform to construct a bacterial strain that is capable of producing higher alcohols. We have also shown the promise of using duckweed as the basis for developing higher alcohols, illustrating that this group of plants represents an ideal fermentation substrate that can be considered the next generation of alternative energy feedstocks.

## Introduction

The worldwide decline of fossil fuel reserves in recent years has created an urgent need for the development of new fuel sources, such as bio-alcohols, biohydrogen, biodiesel and the recovery of biomass waste heat [1-5]. Such initiatives to produce alternative energy from renewable materials have the added benefit of addressing the numerous pollution problems that result from fossil fuel consumption [6]. The fermentation of carbohydrates is a well-known way to produce biofuels, and the value-added fermentation process is also attractive for environmental reasons [3]. An appealing cost-effective approach is the use of the abundant, surplus agricultural waste or by-products that represent a source of renewable materials for the production of biofuel [7]. There have been great progresses recently in the production of higher alcohols that have high vapor pressure, low hygroscopicity and high energy density, including isobutanol, 2-methyl-1-butanol, octyl alcohol, 3-methyl-1-butanol, and long carbon–chain enol [8].

Higher alcohols have been produced from microorganisms that are used as microbial “cell factories”, by improving them with bioengineering traits from other organisms including various yeast species, *E.coli* [9,10], *Corynebacterium glutamicum* [1], and *Clostridium spp* [11]*.* Such efforts have yielded promising results by replacing the traditional biofuel ethanol with new alternatives, through the use of metabolic engineering to transform metabolic pathways in microorganisms that produce alcohols, or to construct new pathways in microorganisms that do not naturally produce higher alcohols [12]. The use of bioengineering tools to develop new biofuels has focused primarily on the development and breeding of artificial microbial strains that produce multiple biofuel compounds, such as terpene [13], alkane, higher alcohols efficiently from the substrate of absolute glucose or pentose [14,15]. Recent efforts to so, combined with efforts to optimize the processes for augmenting yields of higher alcohols from glucose, have involved a significant amount of work. However, there has been little success in using bioengineered strains to directly ferment hydrolysates of surplus agricultural waste or plant by-products to produce higher alcohols on an industrial scale. This poses a great challenge to the increased use of non-edible biomass feedstocks as the generation of biofuels. In particular, there are no published assessments of the use of bioengineered microbe strains to directly ferment non-food crop sources, such as duckweed, to produce higher alcohols.

The duckweed family (*Lemnaceae*) is a group of fast-growing, floating aquatic species that represent the world’s smallest and simplest flowering plants. They have been proposed as an inexpensive, sustainable source of plant biomass for producing biofuels. There are many advantages to using duckweed in this process. For instance, a maximum yield of biomass is obtained in a short time with minimum costs. Its use does not detract from food supplies for human populations, and they can be harvested more easily than algae and other aquatic plants. Compared to other starch feedstocks such as corn and cassava, it is easy to grind, and minimal energy is needed to pretreat it [16,17]. Some recent studies have used duckweed as fermentation substrates for the production of ethanol by *Saccharomyces cerevisiae* [18,19]*.* Current studies have used genetic engineering to remould microorganisms, such as mutant strains of yeast and *E.coli*, to ferment the hydrolysates of duckweed to produce alcohol, but yields were very low [20]. Therefore, in order to further develop methods for the use of duckweed for the production of higher alcohols, we aimed to develop a microorganism that functions as the better host of bioengineered strains to pair with the fermentation substrates of duckweed.

Although *E. coli* has proven to be a good expression host in some respects, its overall performance has been hampered by poor tolerance for the end-product, compared to that of coryneform bacteria or fungi such as yeasts, and by poor resistance to the toxic compounds that are found in fermentation substrates after pretreatment processes [21-24]. Therefore, increased attention has been directed at the potential for corynebacterium to possibly act as a better host.

*Corynebacterium crenatum* is a fast-growing, aerobic, gram-positive, non-sporulating coryneform bacterium. It is often used to produce large quantities of some long-chain amino acids such as leucine, isoleucine and arginine for industrial applications [25-27]. For example, the *C. crenatum* strain SYPA 5–5 (CGMCC No. 0890) of producing 32.0 g/L of L-arginine was obtained by random chemical mutagenesis [28]. The mutant strain *C. crenatum* AS 1.1004 was created by nitrosoguanidine continuous mutagenesis and can also produce L-leucine of 20 g/L up to large-scale industrial application [26]. The terminal feedback control function in another mutant strain, *C. crenatum* AS 1.998, was relieved and no longer subject to inhibition by isoleucine, resulting in yields of up to 14 g/L [27]. *Corynebacterium glutamicum*, however, is usually used to produce short-chain amino acids(< C_6_), such as glutamic acid, valine, lysine in industrial scale production, rather than long-chain amino acids leucine, isoleucine. In this regard, these studies showed that this bacterium has great potential in terms of the production of long-chain amino acids, therefore, signified it may be provide a distinct advantage as an effective “cell factory” for the synthesis of C_5–6_ higher alcohols via metabolic engineering such as 2-methyl-1-butanol, 3-methyl-1-butanol and 3-methyl-1-pentanol. Because these higher alcohols involves some common precursors with amino acids in 2-keto acid pathways, therefore, *C. crenatum* has shown potential for use in the production of higher alcohols. In this paper, we reported the first use of *C. crenatum* as an expression host to produce higher alcohols via the use of metabolic engineering to construct strains for that purpose. We also investigated the potential of producing higher alcohols using the hydrolysates of duckweed as fermentation substrates. Our findings provide valuable, fundamental insights that can facilitate further development of methods to use duckweed in the production of higher alcohols as biofuels.

## Materials and methods

### Experimental design

**Figure 1:**
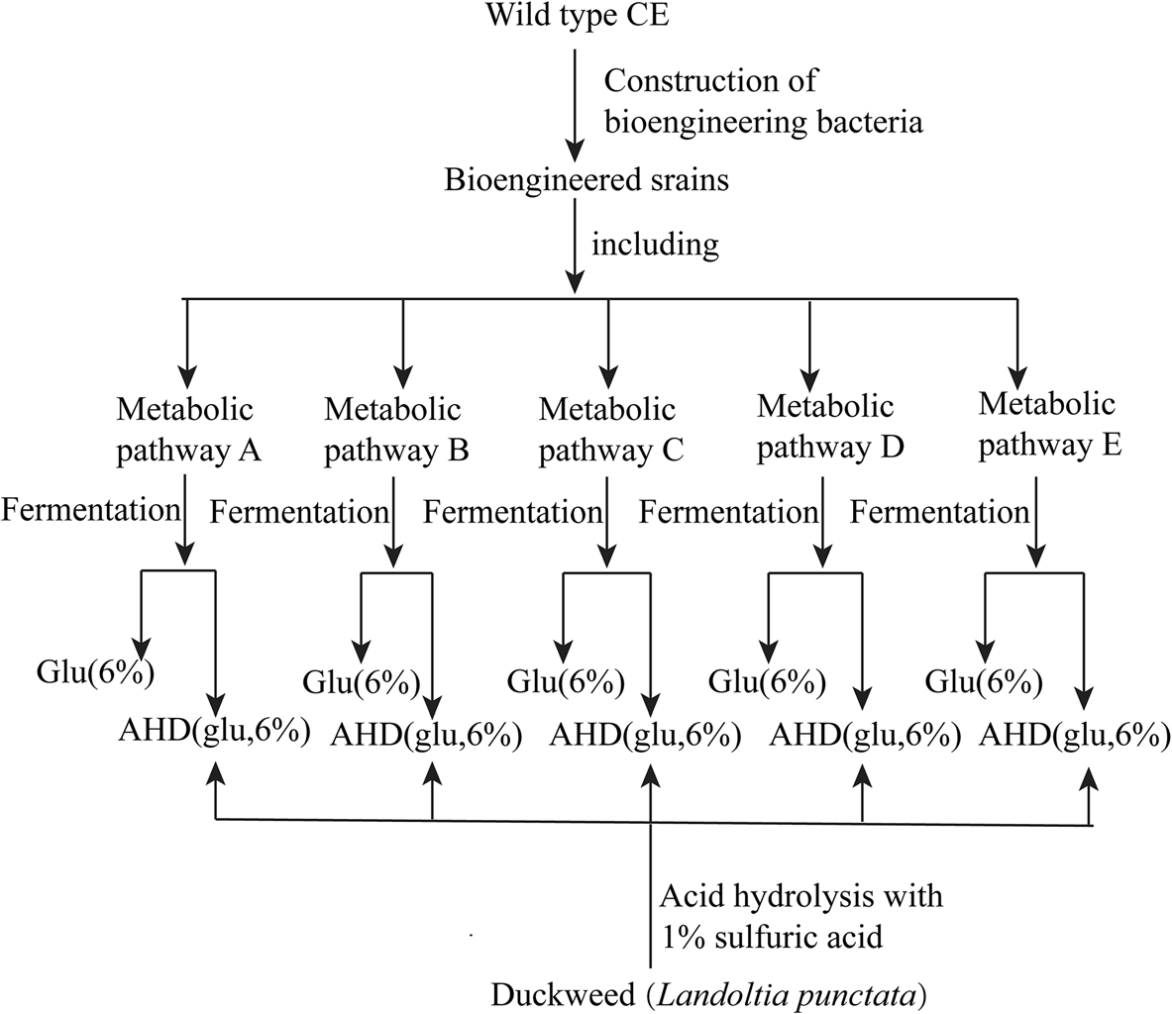
**Flowsheet of the design for experimental processes.** CE: *C. crenatum* CICC 20153; Glu: glucose; AHD: acid hydrolysates of duckweed as the fermentation substrate, meaning the products resulting from pretreatment of duckweed with acid hydrolysis.

The purpose of this experiment was to investigate the potential of producing higher alcohols from duckweed, by building an exogenetic metabolic pathway using a “non-natural” corynebacterium to produce alcohols, *C. crenatum*. Glucose and duckweed hydrolysate were used as the two fermentation substrates. Experiments were conducted following the methodology illustrated in the flowsheet of fermentation processes (Figure 1). Three replicates of each experiment and assay were conducted, each in a 150-mL triangular flask, unless otherwise indicated.

### Bacterial strains, media, and growth conditions

All plasmids were propagated using *E. coli* DH5α competent cells (Takara: 9057, Chengdu, China). The *E. coli* DH5α bacterial strains were cultivated in LB medium, and grown at 37°C in a rotary shaker for 4 h. The bacterium *C. crenatum* CICC 20135 was purchased from the China Center of Industrial Culture Collection (CICC, Beijing, China), and rejuvenated and propagated in a nutrition gravy medium (peptone 5.0 g, beef extract 3.0 g, NaCl 5.0 g, glucose 20 g, ddH2O 1 L, pH 7.0) at 30°C in a rotary shaker for 72 h. Cultures of *Lactococcus lactis cremoris* CICC 1605 were purchased from the CICC and cultivated in MRS medium (peptone 10 g, beef extract 3.0 g, yeast extract 3.0 g, K2HPO4 2 g, citric acid diamine 2 g, sodium acetate 2 g, glucose 20 g, MgSO_4_ · 7H_2_O 0.6 g, MnSO_4_ · 4H_2_O 0.25 g, ddH2O 1 L, pH 6.2) at 32°C in a rotary shaker for 72 h. Cultures of *Saccharomyces cerevisiae* AH109 were purchased from Clontech Laboratories, Inc. (Beijing, China) and reproduced at 30°C in TGY medium in a rotary shaker for 72 h. All cultures of the bioengineered strains of *E. coli* and *C. crenatum* CICC 20135 were then induced with 2 mM isopropyl-β-D-thiogalactoside (IPTG) and grown at 30°C for 18 h. Antibiotics (ampicillin, 100 μg/mL; chloramphenicol, 35 μg/mL; kanamycin, 50 μg/mL) were added, as needed. The bioengineered strains were reproduced in a rotary shaker under the following conditions: *E. coli* in LB medium at 37°C for 12 h, and *C. crenatum* in a nutrition gravy medium at 30°C for 48 h, and then moved into 4°C to terminate reaction.

### Construction of engineered strains of *Corynebacterium crenatum* CICC 20135

All Restriction enzymes were purchased from NEB (Shanghai, China) and T4 DNA ligase (EL0334) was supplied by MBI Fermentas (Chengdu, Beijing). Oligonucleotides were ordered from BGI (Beijing, China). The strains of *C. crenatum* CICC 20135 were constructed as host strains. All strains and plasmids used in the study were listed in Table 1, and the oligonucleotides were listed in Table 2.

**Table 1:** **The bacterial strains and vectors used in the bioengineering of bacteria to produce higher alcohols**

**Strain or plasmid**	**Relevant genotype**	**Source**
Strains		
DH5α	F^−^, φ 80d*lacZ* ΔM15, Δ(*lacZYA*-*argF* )U169, *deoR*, *recA1*, *endA1*, *hsdR17*(*rK* ^−^, *mK* ^*+*^), *phoA*, *supE44*, *λ* ^*−*^,*thi* ^*−1*^, *gyrA96*, *relA1*	Takara: 9057
*C. crenatum*	*ompT, hsdSB (rB* ^*−*^ *mB* ^*−*^ *),gal, dcm*	CICC 20153
Plasmids		
pSTV29	pACYC184 ori; Cm^r^; PLlacO^−1^: MCS	Takara: 3332
p29-pBL	pBL1ori; Cm^r^; PLlacO^−1^: MCS	This study
PEC-XK99E	pGA1 Km^r^ pTrc99A MCS P-trc,lacI^q^	From CAS
pXMJ19	pBL1ori; Km^r^; MCS P-trc, lacI^q^	From CAS
PEC-KA	pGA1 Km^r^ pTrc99A MCS P-trc, lacI^q^: *Kivd*(LL)-*ADH2*(SC)	This study
p29- AL	pBL1ori; Cm^r^; PLlacO-1:MCS:*LEU2*(SC)	This study
p29- ALI	pBL1ori; Cm^r^; PLlacO-1: *LEU2*(SC)-*ILV2*(SC)	This study
p29- ALII	pBL1ori; Cm^r^; PLlacO-1: *LEU2(SC)-ILV2-ILV5*(SC)	This study
p29- BI	pBL1ori; Cm^r^; PLlacO-1: *ILV2*(SC)	This study
p29- BII	pBL1ori; Cm^r^; PLlacO^−1^: *ILV2*(SC)-*ILV5*(SC)	This study
p29- BIII	pBL1ori; Cm^r^; PLlacO^−1^: *ILV2*(SC)-*ILV5*(SC)-*ILV3*(SC)	This study
p29- CB	pBL1ori; Cm^r^; PLlacO^−1^: *BAT2*(SC)	This study
p29-DL	pBL1ori; Cm^r^; PLlacO^−1^: *LEU4*(SC)	This study
p29-DLL	pBL1ori; Cm^r^; PLlacO^−1^:*LEU4*(SC)-*LEU1*(SC)	This study
p29-FL	pBL1ori; Cm^r^; PLlacO^−1^: *LEU1*(SC)	This study

SC: *S. cerevisiae* AH109; LL: *Lactococcus lactis subsp. cremoris* CICC 1605.

**Table 2:** **Primers used in the bioengineering of bacteria to produce higher alcohols**

**Number**	**Primers name**	**Primer 5′-3′**
A1	LEU2-S	ACATGCATGCGGATGTCTGCCCCTAAGAAGAT
	LEU2-AS	ACGCGTCGACTTAAGCAAGGATTTTCTTAACTTC
A2	ILV2-S	ACGCGTCGACAAGGAGCCAGATGATCAGACAATCTACGCT
	ILV2-AS	CGCGGATCCTCAGTGCTTACCGCCTGTAC
A3	ILV5-S	CGCGGATCCAAGGAGGCCTCATGTTGAGAACTCAAGCCGC
	ILV5-AS	GCGAGCTCTTATTGGTTTTCTGGTCTCAACTT
B1	ILV2-S	AAAACTGCAGATGATCAGACAATCTACGCT
	ILV2-AS	ACGCGTCGACTCAGTGCTTACCGCCTGTAC
B2	ILV5-S	ACAGGTCGACAAGGAGGTCATGTTGAGAACTCAAGCCGC
	ILV5-AS	CGCGGATCCTTATTGGTTTTCTGGTCTCAA
B3	ILV3-S	CGCGGATCCAAGGAGCTGCATGGGCTTGTTAACGAAAGT
	ILV3-AS	GCGAGCTCTCAAGCATCTAAAACACAACC
C1	BAT2-S	CGGCCTGCAGGATGACCTTGGCACCCCTAGA
	BAT2-AS	CGCGGATCCTCAGTTCAAATCAGTAACAA
D2	LEU1-S	TCCCCCCGGGAAGGAGACTAATGGTTTACACTCCATCCAA
	LEU1-AS	GCGAGCTCCTACCAATCCTGGTGGACTTT
C2	leu1-2as	CGCGGATCCAAAGGAGGCCGCATGGTTTACACTCCATCCAAGG
	leu1-2s	TCCCCCCGGGCTACCAATCCTGGTGGACTTT
C3	leu2-2s	TCCCCCCGGGAAAGGAGGCCGCATGTCTGCCCCTAAGAAGATC
	leu2-2as	GCGAGCTCTTAAGCAAGGATTTTCTTAAC
D1	leu4-2as	CGGCCTGCAGGATGGTTAAAGAGAGTATTAT
	leu4-2s	TCCCCCCGGGTTATGCAGAGCCAGATGCCG
D2	LEU1-S	TCCCCCCGGGAAGGAGACTAATGGTTTACACTCCATCCAA
	LEU1-AS	GCGAGCTCCTACCAATCCTGGTGGACTTT
F1	leu1-2 s	CGGCCTGCAGGATGGTTTACACTCCATCCAA
	leu1-2as	CGCGGATCCCTACCAATCCTGGTGGACTT
G1	pBL1-as	TCCCCGCGGATTCGGGGTCGTTCACTGGT
	pBL1-s	CCATCGATAACAACAAGACCCATCATAG
K1	Kivd-s	AAAACTGCAGATGTATACAGTAGGAGATTACCT
	Kivd-as	GCTCTAGATTATGATTTATTTTGTTCAGC
H1	ADH2-s	GCTCTAGAAGGAAACTCAATGTCTATTCCAGAAACTCAA
	ADH2-as	CGGGGTACCTTATTTAGAAGTGTCAACAACG

s = sense and as = antisense.

**Figure 2:**
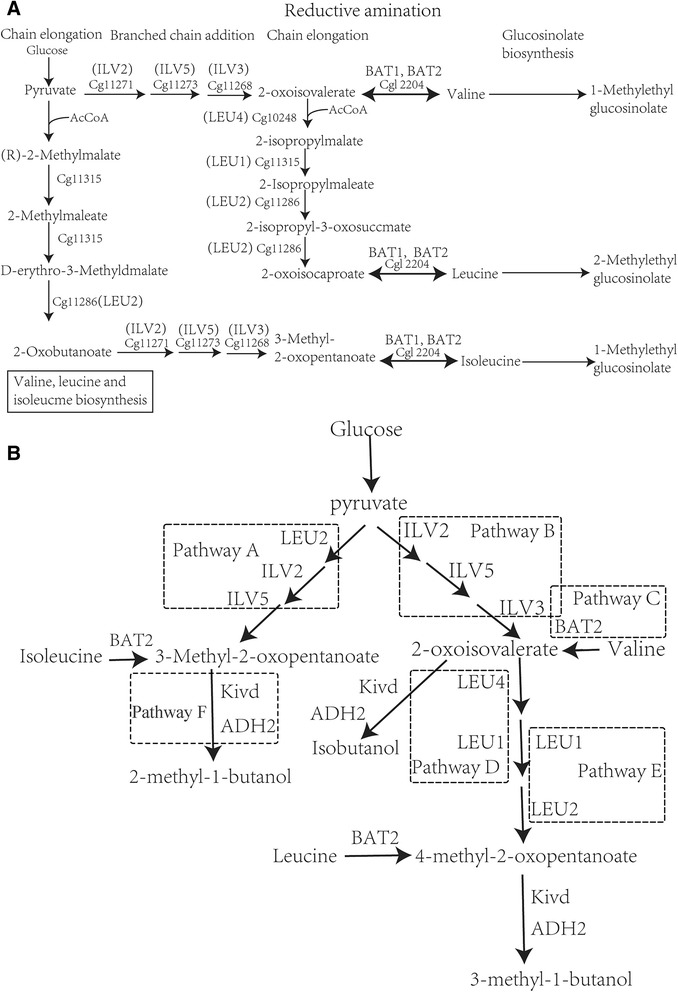
**The metabolic pathways of 2-keto acid compounds and construction of the biological synthesis pathways in bioengineered strains for producing higher alcohols via fermentation by microbes. A**: the common schematic pathways of *S. cerevisiae* (2-Oxocarboxylic acid metabolism: http://www.genome.jp/dbget-bin/www_bget?pathway:sce01210) and *C. crenatum* for producing 2-keto acid compounds. The genes *LEU2*, *ILV5*, *ILV2*, *ILV3*, *LEU4*, *LEU1*, *BAT1* and *BAT2* play a role in the synthesis of 2-keto acids in *S. cerevisiae*. The genes Cgl 1315, Cgl 1286, Cgl 1271, Cgl 1273, Cgl 1268, Cg1 0248 and Cgl 2204 act as catalysts in the production of 2-keto acids in *C. crenatum. LEU2*, Cgl 1286:3-isopropylmalate dehydrogenase. *ILV5*, Cgl 1273:ketoacid reductiove isomerase. *ILV2*, Cgl 1271:acetyl lactic acid synthase. *ILV3*, Cgl 1268: dihydroxy acid dehydratase. *LEU4*, Cg1 0248:2-isopropyl malic acid synthase. *LEU1*, Cgl 1315: 3-isopropylmalate dehydratase. *BAT1*, *BAT2*, Cgl 2204: branched-chain amino acid transaminase. **B**: construction of the metabolic pathways for higher alcohol production in *C. crenatum* based on 2-Keto acid pathways; ADH2: Alcohol dehydrogenase; Kivd: alpha-ketoisovalerate decarboxylase. Metabolic pathway A: *LEU2-ILV2-ILV5* + *KIVD-ADH2*; Metabolic pathway B: *ILV2-ILV5-ILV3* + *KIVD-ADH2*; Metabolic pathway C: *BAT2* + *KIVD-ADH2*; Metabolic pathway D: *LEU4-LEU1* + *KIVD-ADH2*; Metabolic pathway E: *LEU1* + *KIVD-ADH2.*

The approach used to construct new metabolic pathways was illustrated in Figure 2B. Five metabolic pathways, labelled A (*LEU2*-*ILV2*-*ILV5*), B (*ILV2*-*ILV5*-*ILV3*), C (*BAT2*), D (*LEU4*-*LEU1*), and E (*LEU1*), were constructed using genes from *S. cerevisiae* AH109. The sixth metabolic pathway, F (*Kivd*-*ADH2*) was constructed using the *Kivd* gene from *L. lactis cremoris* CICC1605 and the *ADH2* gene from *S. cerevisiae* AH109.

The genes *LEU2*, *ILV2*, and *ILV5* of metabolic pathway A were amplified with the primer pairs A1, A2, and A3, respectively. The genes *ILV2, ILV5* and *ILV3* of pathway B were amplified with the primer pairs B1, B2 and B3, respectively. The gene *BAT2* was amplified with the primer pair C1. The genes *LEU4* and *LEU1* of metabolic pathway D were amplified with the primer pairs D1 and D2. The genes *Kivd* and *ADH2* of metabolic pathway F were amplified with the primer pairs F1 and F2. The gene *LEU1* of pathway E was amplified with the primer pair D2, and finally the gene pBL1was amplified with the primer pair G1. The genes *Kivd* and *ADH2* were amplified with the primer pairs K1, and H1, respectively.

The plasmids p29-pBL, PEC-KA, p29-AL, p29-ALI, p29-ALII, p29-BI, p29-BII, p29-BIII, p29-CB, p29-DL, p29-DLL, p29-FL and p29-FLL were constructed as follows. The gene *pBL1* from the vector pXMJ19 was inserted into vector pSVT29 using the restriction enzymes sacII and *Cla*I to construct plasmid p29-pBL. The in-series genes *KIVD-ADH2* were inserted into vector PEC-XK99E using restriction enzymes *Pst*I, *Xba*I, *Kpn*I to construct plasmid PEC-KA. The in-series genes *LEU2-ILV2-ILV5* were inserted into vector p29-pBL using restriction enzymes *sph*I, *Sal*I, *Bam*HI, *sac*I and T4 ligase to construct plasmids p29-AL, p29-ALI and p29-ALII. The in-series genes *ILV2-ILV5-ILV3* were inserted into vector p29-pBL using restriction enzymes *pst*I, *Sal*I, *Bam*HI, *sac*I and T4 ligase to construct plasmids p29-BI, p29-BII, p29-BIII. The gene *BAT2* was inserted into vector p29-pBL using restriction enzymes *sbf*I, *Bam*HI and T4 ligase to construct plasmid p29-CB. The in-series genes *LEU4-LEU1* were inserted into vector p29-pBL using restriction enzymes *sbf*I, *xma*I, *sac*I and T4 ligase to construct plasmids p29-DL and p29-DLL. The gene *LEU1* was inserted into vector p29-pBL using restriction enzymes *sbf*I, *Bam*HI and T4 ligase to construct plasmid p29-FL. The ribosome binding site (RBS) sequence was inserted at the position 6–8 nucleotides upstream of each structural gene to facilitate mRNA translation.

The preparation of competence cells of *C. crenatum* was described in the Handbook of *Corynebacterium glutamicum* [29]. All construction plasmids were introduced into *C. crenatum* using the electroporation protocol [30,31]. Electroporation was conducted as 100Ω, 50 μF, 2.2 kV, 8 ms with Gene Pulser Xcell Microbial System165–2662 (BIO-RAD, Chengdu).

### Genes expression analysis

Genes expression of all recombinant genes for each metabolic pathway in the production host were analyzed using semi-quantitative RT-PCR. RNA extraction and cDNA synthesis were prepared according to operation manual of TransScript First-Strand cDNA Synthesis SuperMix kit of the manufacturer (TransGen Biotech, Beijing, China). Semi-quantitative RT-PCR analysis: Expression profile of all recombinant genes were evaluated based on semi-quantified analysis system using Gel-Pro analyzer 4.0 software (Media Cybernetics, Silver Spring, MD), the calculated result of all exogenous genes expression was indicated using integral optical density (IOD).

### Pretreatment processing of dried duckweed

Duckweed (wild *Landoltia punctata*) was collected opportunistically from the surface of wild ponds in Huilong Town, Xinjin County, Chengdu, China. The fresh plants were dried at 60°C with drying oven(DHP9050A, Shanghai, China), crushed into powder with (FS100S-3, Guangzhou, China), and then hydrolyzed with 1% H_2_SO_4_. The starch content was calculated based on the total sugar content (starch content = glucose content × 0.91) [32,33]. The crude protein content was measured as CP = K_j_ N × 6.25 [34,35]. Cellulose content was measured with spectrophotometry: a 10-g sample of duckweed was placed in 1 L water, 60 mL of 60% H_2_SO_4_ were added, and the plants were left to decompose for 30 min. We added 2% anthrone reagent (v/v) to the hydrolyzed mixture, left it for 2 min, and then measured the absorbance at 620 nm [36-38]. We then calculated the cellulose content of samples according to a standard curve, using the formula: cellulose content Y (%) of duckweed = X (cellulose content of standard sample) × a (diluted multiples) × 100/W (total weight of samples). The content of lignin was determined using acetyl bromide according to standard methods [39,40]. The resulting components of duckweed were listed in Table 3.

**Table 3:** **The main components and carbohydrate composition after pretreatment with acid hydrolysis of wild duckweed** ***Landoltia punctata*** **(before fermentation: 0 h)**

**Pretreatment sample**	**Cellulose content**	**Protein content**	**Starch content**	**Lignin content**	
**Main components**					
Dried duckweed	26.64%	23.71%	31.31%	1.89%	
Fresh duckweed	3.81%	3.12%	4.32%	0.038%	
**Carbohydrate production after acid hydrolysis**					
Pretreatment sample	Glucose	xylose	Galactose	Fructose	Arabinose
Dried duckweed^*a*^	5.22 ± 0.28	1.03 ± 0.055	0.49 ± 0.088	0.72 ± 0.21	0.46 ± 0.24
Fresh duckweed^*a*^	0.61 ± 0.14	0.094 ± 0.11	0.044 ± 0.013	0.121 ± 0.016	0.094 ± 0.007

Content (g) of various components measured in 10 g pretreatment samples. ^*a*^Acid hydrolysis pretreatment method.

Duckweed substrates were pretreated using established methods of acid hydrolysis [20]. The products of hydrolysis were then fermented by bioengineeried strains of *C. crenatum*. The initial total glucose content of the pretreated duckweed was adjusted to 60 ± 2.61 g/L, and the pH of hydrolysates was adjusted to 6.7 using 0.1% Ca(OH)_2_. The liquefied hydrolysates of duckweed were then used as the substrate for further fermentation experiments; their carbohydrate composition (i.e., after pretreatment, at 0 h of fermentation) was presented in Table 3.

### Fermentation conditions and process

In order to evaluate the effect of using different fermentation substrates, the experiments were divided into two parts: part one used the traditional substrate of glucose, to provide a basis for comparison, and part two was conducted using the hydrolysates of duckweed.

The fermentation substrate in glucose trials was 60 g/L glucose, combined with a Trace Metals Mix solution (NaCl 5 g/L, (NH4)_2_SO_4_ 20 g/L, KH_2_PO_4_ 1.5 g/L, MgSO_4_ · 7H_2_O 0.3 g/L, FeSO_4_ · 7H_2_O 0.05 g/L, MnSO_4_ · H_2_O 0.01 g/L, CH_3_COONH 1.0 g/L) that contained 10 g/L peptone and 5 g/L yeast extract.

The fermentation substrate in duckweed trials was hydrolysates of duckweed in 20 mL of modified M9 medium (2 g (NH_4_)_2_PO_4_, 2 g KH_2_PO_4_, 1 g K_2_HPO_4_, 1 g NH_4_Cl, 0.5 g NaCl, 0.5 mM MgSO_4_, 1 mM CaCl_2_, 20 mg vitamin B1, and 2 mg biotin per L of water) containing 5 g/L yeast extract, 10 g/L peptone and Trace Metals Mix solution (2 g H_3_BO_3_, 2.1 g MnCl_2_ · 4H_2_O, 0.3 g ZnSO_4_ · 7H_2_O, 0.002 g MnSO_4_ 2.5 g, Na_2_MoO_4_ · 2H_2_O, 0.05 g CuSO_4_ · 5H_2_O, 21.2 mg Co(NO_3_)2 · 6H_2_O, and 0.05 g FeSO_4_ per L of water) in a 150-mL triangular flask.

The substrates were fermented under aerobiotic condition; and 2 mL of a rejuvenated seeding solution from liquid medium was inoculated into 50 mL of the acid hydrolysates in a 150 mL triangular flask. All cultures were induced with 2 mM IPTG, kanamycin, and chloramphenicol, kept at 30°C to allow 4 h of growth, and then fermented at 30°C with shaking at 200 rpm for 96 hours in a constant-temperature oscillation incubator. The initial pH values of fermentation substrates were adjusted to 7.0.

### Detection of alcohols produced from fermentation

Alcohol compounds were measured with a model 6890 gas chromatograph (GC) equipped with a flame ionization detector (Agilent Technologies, Santa Clara, CA, USA) with a model 7673A automatic injector, sampler, and controller (Hewlett-Packard). Alcohol compounds were separated out using a ZB-WAX capillary column (30 m, 0.25 mm inside diameter, 0.25 μm film thickness; Phenomenex Inc., PA, USA). The GC oven temperature was held initially at 40°C for 5 min, and then raised stepwise, by 15°C/min, until it reached 150°C. It was then raised by 50°C/min up to 250°C, and held for 4 min. Helium was used as the carrier gas, with an inlet pressure of 9.3 lb/in^2^. The injector and detector were maintained at 220°C. A 1-μL volume of supernatant from the culture broth was injected in split-injection mode at a 1:30 split ratio. For other secreted metabolites, the constituent compounds (20 μL) were detected with an Agilent 1100 high-performance liquid chromatography system equipped with an auto-sampler and a Bio-Rad (Hercules, CA: carbohydrate analysis column Aminex HPX-87P Column 300×7.8 mm catalog 125–0098 serial 426070) (5 mM H_2_SO_4_, 0.6 mL/min; column temperature at 65°C). Glucose was detected with an ELSD 2000 CSC detector, while organic acids were detected using a photodiode array detector at 210 nm. Concentrations were determined using extrapolation from standard curves.

Inhibitors: glucuronic acid, p-coumaric acid, syringic acid, ferulic acid were determined with a DIONEX UltiMate 3000 liquid chromatograph in a column packed with Aminex HPX-87H and 0.05 mM H_2_SO_4_ on Chromosorb WAW. The chromatography was conducted at an injector temperature of 175°C, detector temperature of 180°C, and oven temperature of 125°C. Determination of furfural and 5-Hydroxymethylfurfural were determined with HPLC according to the methods [41,42].

### Statistical analysis

For each experiment and assay, we calculated the mean response variables and their standard deviation (SD), unless otherwise indicated. Comparisons of variable(s) were made with Student’s t-test; values of P < 0.05 were considered to indicate significant differences. Multiple comparisons were made using the LSD. Tukey’s honest significant difference test was used when the null hypothesis was rejected (P < 0.05). Statistical analyses were conducted using the software program SPSS 21.0 (IBM, USA).

## Results

**Figure 3:**
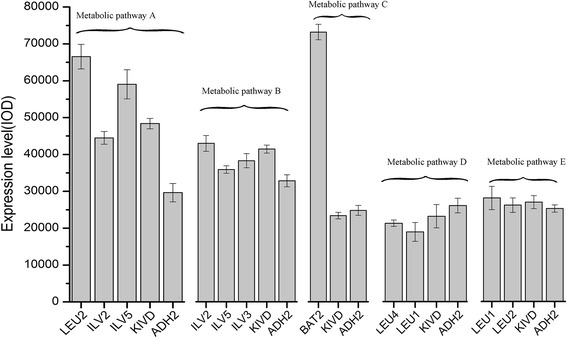
**Genes expression of all recombinant genes for each metabolic pathway in the production host were analyzed using semi-quantitative RT-PCR.** Semi-quantitative RT-PCR analysis: Expression profile of all recombinant genes were evaluated using Gel-Pro analyzer 4.0 software.

In this study, the interrelated genes that control the metabolic pathways of isoleucine, leucine and valine (Figure 2A) were overexpressed into the host *C. crenatum* in order to enable it to produce higher alcohols. First, the success of all exogenous genes expression were confirmed by RT-PCR assay. The results of RT-PCR assay were further semi-quantitatively estimated using Gel-Pro Analyzer 4.0 software (Figure 3). Our results demonstrated that the expression quantity of recombinant genes for each metabolic pathway remained relatively different at same induction condition. We then investigated the potential of the bioengineered strains to produce higher alcohols from the hydrolysates of duckweed, as a way to use the plant as feedstock for a source of alternative energy. To verify whether the bioengineered strains were able to produce higher alcohols at all, we first investigated their potential using 60 g/L of glucose as the fermentation substrate for 96 h.

**Figure 4:**
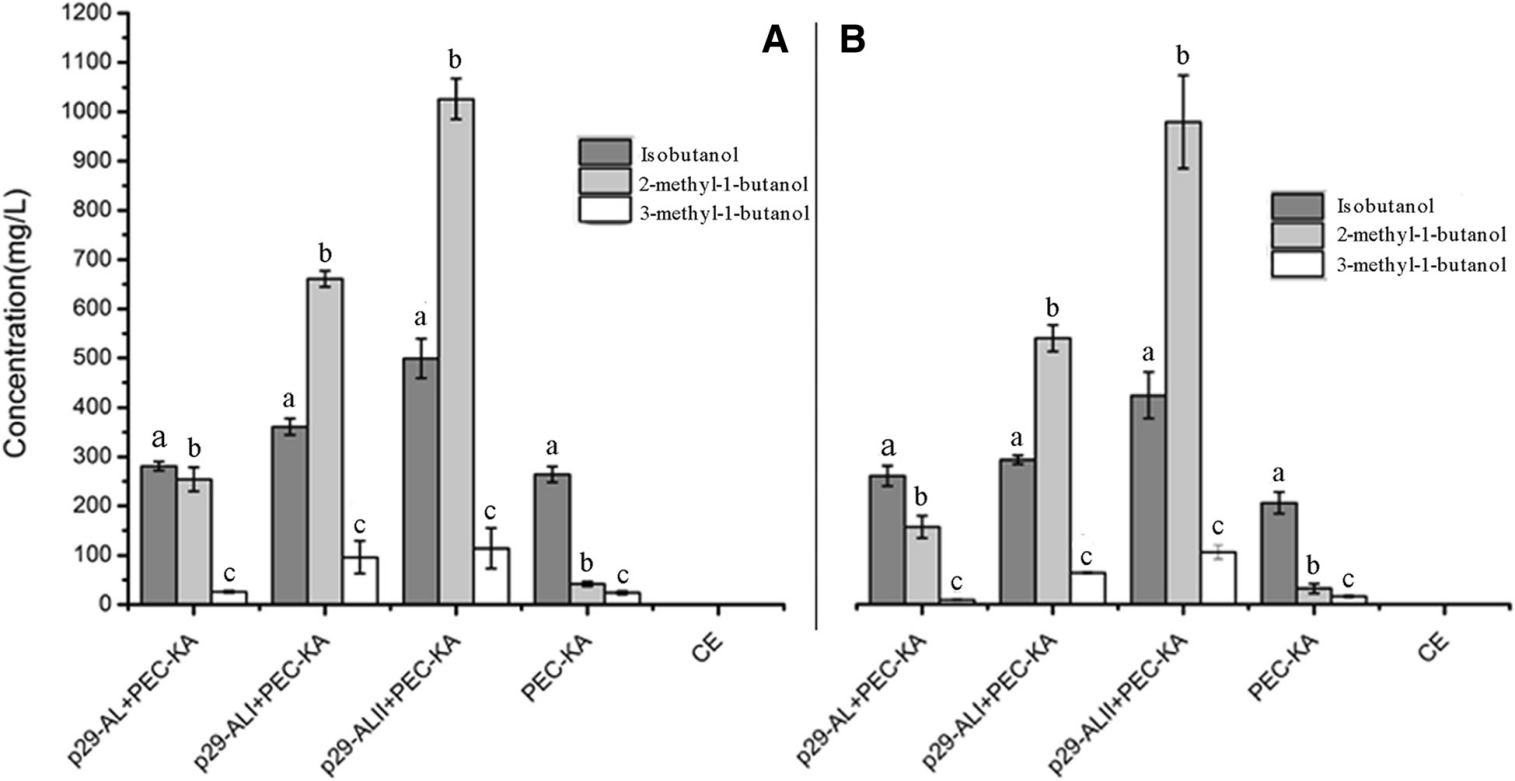
**Production of higher alcohols via construction of metabolic pathway A in the bioengineered strain of** ***C. crenatum*** **.** (4**A**) Yield of higher alcohols from glucose. (4**B**) Yield of higher alcohols from the acid hydrolysates of duckweed. p29-AL + PEC-KA: *LEU2* + *KIVD*-*ADH2*; p29-ALI + PEC-KA: *LEU2-ILV2* + *KIVD-ADH2*; p29-ALII + PEC-KA: *LEU2-ILV2-ILV5* + *KIVD-ADH2*. CE: *C. crenatum* CICC 20153. Error bars indicate +/− SD (n = 3). The different letters indicate significant differences based on multiple comparisons for different alcohol products (*P* < 0.05).

The fermentation results achieved using metabolic pathway A (mpA) that we constructed are shown in Figure 4. The highest yield of 2-methyl-1-butanol (1026.61 mg/L) was obtained when three serial genes *LEU2-ILV2-ILV5* (mpA, p29-ALII) were overexpressed in *C. crenatum* using glucose as the fermentation substrate (Figure 4A, Table 4). Similarly, the bioengineered strain with those three genes produced the highest yield of 2-methyl-1-butanol (981.79 mg/L) from the hydrolysates of duckweed. However, the yield was clearly lower in the pathways that contained two serial genes (p29-ALI), dropping to 661.79 mg/L with glucose (Figure 4A) and to 541.13 mg/L with duckweed (Figure 4B). The yield of 2-methyl-1-butanol obtained when the bioengineered strain had only one overexpressed gene (p29-AL) was approximately 200 mg/L. We also investigated changes in the production of isobutanol and 3-methyl-1-butanol. Even when only one gene (*LEU2*) was overexpressed, the 2-methyl-1-butanol yield improved noticeably, while the yields of isobutanol and 3-methyl-1-butanol did not improve (Figure 4). Thus, the yields of three kinds of high alcohols had improved to a certain extent when the serial genes *LEU2-ILV2* were overexpressed. In particular, the yield of 2-methyl-1-butanol increased significantly when the three genes in series *LEU2-ILV2-ILV5* were overexpressed, but the yield of isobutanol did not increase.

**Table 4:** **The highest yield of higher alcohols produced via bioengineered strains corresponding to relevant metabolic pathways**

**Bioengineered strains**	**Isobutanol (mg/L)**	**2-methyl-1-butanol (mg/L)**	**3-methyl-1-butanol (mg/L)**
Strain with p29-BIII + PEC-KA	1264.63 _a_^*a*^/1154.83 _a_^*b*^	589.43_a_^*a*^/492.76 _a_^*a*^	117.27 _a_^*a*^/101.62 _a_^*b*^
Strain with p29-ALII + PEC-KA	499.31 _b_^*a*^/425.71 _b_^*b*^	1026.61 _b_^*a*^/981.79 _b_^*b*^	114.86 _a_^*a*^/106.24 _a_^*b*^
Strain with p29-DLL + PEC-KA	306.57 _c_^*a*^/228.49 _c_^*b*^	234.76 _c_^*a*^/118.63 _c_^*b*^	748.35 _b_^*a*^/684.79 _b_^*b*^

^*a*^the highest yield from glucose; ^*b*^the highest yield from acid hydrolysates of duckweed.

_a,__b,__c_different letters in table indicate significant differences based on multiple comparisons (*P* < 0.05).

**Figure 5:**
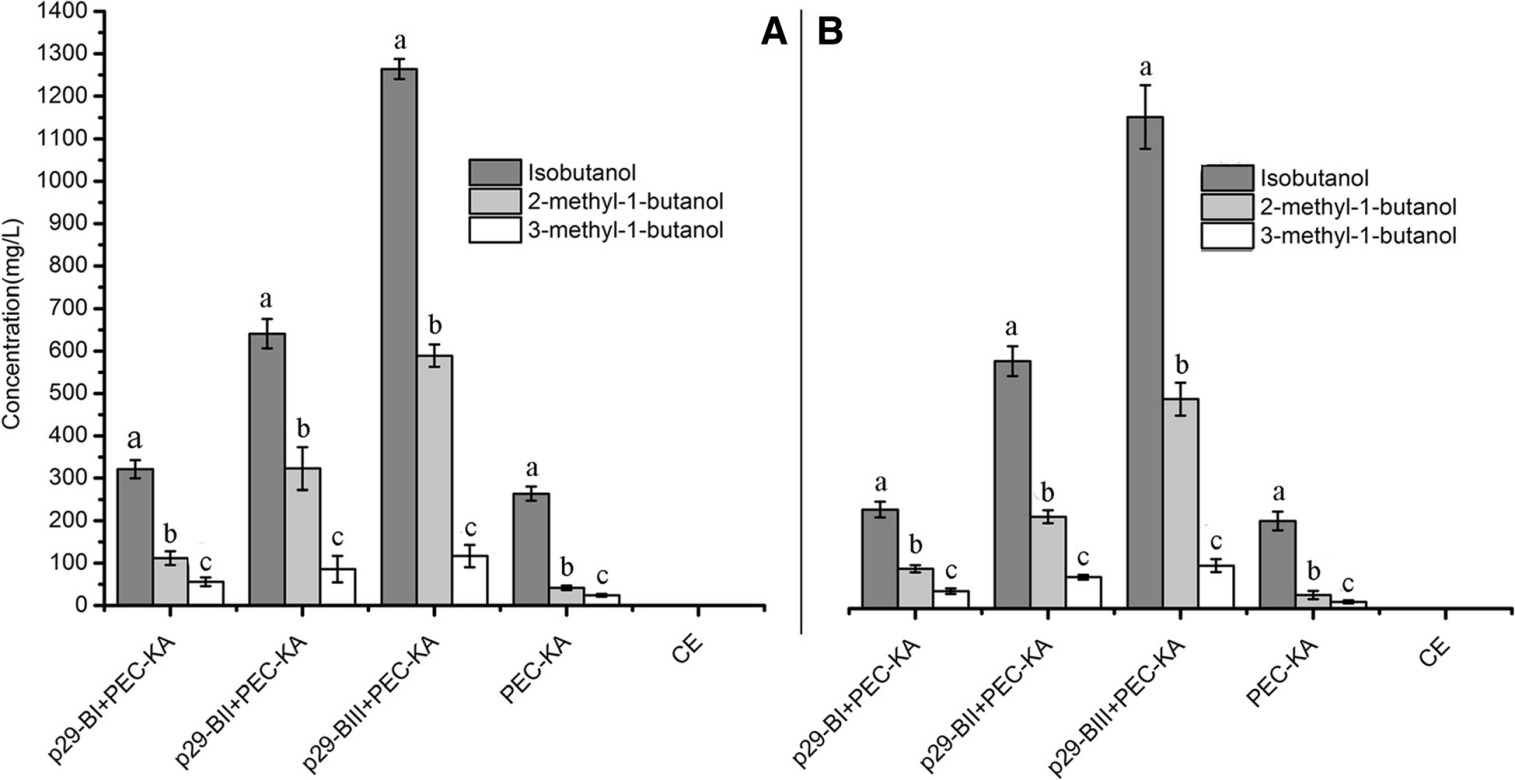
**Production of higher alcohols via construction of metabolic pathway B in the bioengineered strain of** ***C. crenatum*** **.** (5**A**) Yield of higher alcohols from glucose. (5**B**) Yield of higher alcohols from the acid hydrolysates of duckweed. Error bars indicate +/− SD (n = 3). The different letters indicate significant differences based on multiple comparisons for different alcohol products (*P* < 0.05). p29-BI + PEC-KA: *ILV2* + *KIVD-ADH2*; p29-BII + PEC-KA: *ILV2-ILV5* + *KIVD-ADH2*; p29-BIII + PEC-KA: *ILV2-ILV5-ILV3* + *KIVD-ADH2*. CE: *C. crenatum* CICC 20153.

Maximum isobutanol yields were achieved with both substrates (1264.63 mg/L from glucose, and 1154.83 mg/L from duckweed) when the bioengineered strain contained three serial genes (metabolic pathway B, p29-BIII). The second highest yields of that product were 641.58 mg/L (Figure 5A, Table 4) and 581.28 mg/L (Figure 5B) achieved with glucose and duckweed substrates, respectively. The bioengineered strain that contained p29-BI (*ILV2*) produced ~300 mg/L of isobutanol, regardless of the fermentation substrate, which did not improve upon the yield of 206.69 mg/L achieved with the strain that contained only the vector PEC-KA. We also noted changes in the production of other higher alcohols such as 2-methyl-1-butanol and 3-methyl-1-butanol. The yields of isobutanol did not obviously improve when only the gene *ILV2* was overexpressed, but the yields of 2-methyl-1-butanol and 3-methyl-1-butanol did improve. Yields of three higher alcohols improved when the two serial genes *ILV2-ILV5* were overexpressed in *C. crenatum*. This result was especially enhanced when the three serial genes *ILV2-ILV5-ILV3* were overexpressed simultaneously: the yield of isobutanol increased, while the yield of 3-methyl-1-butanol did not improve (Figure 5).

**Figure 6:**
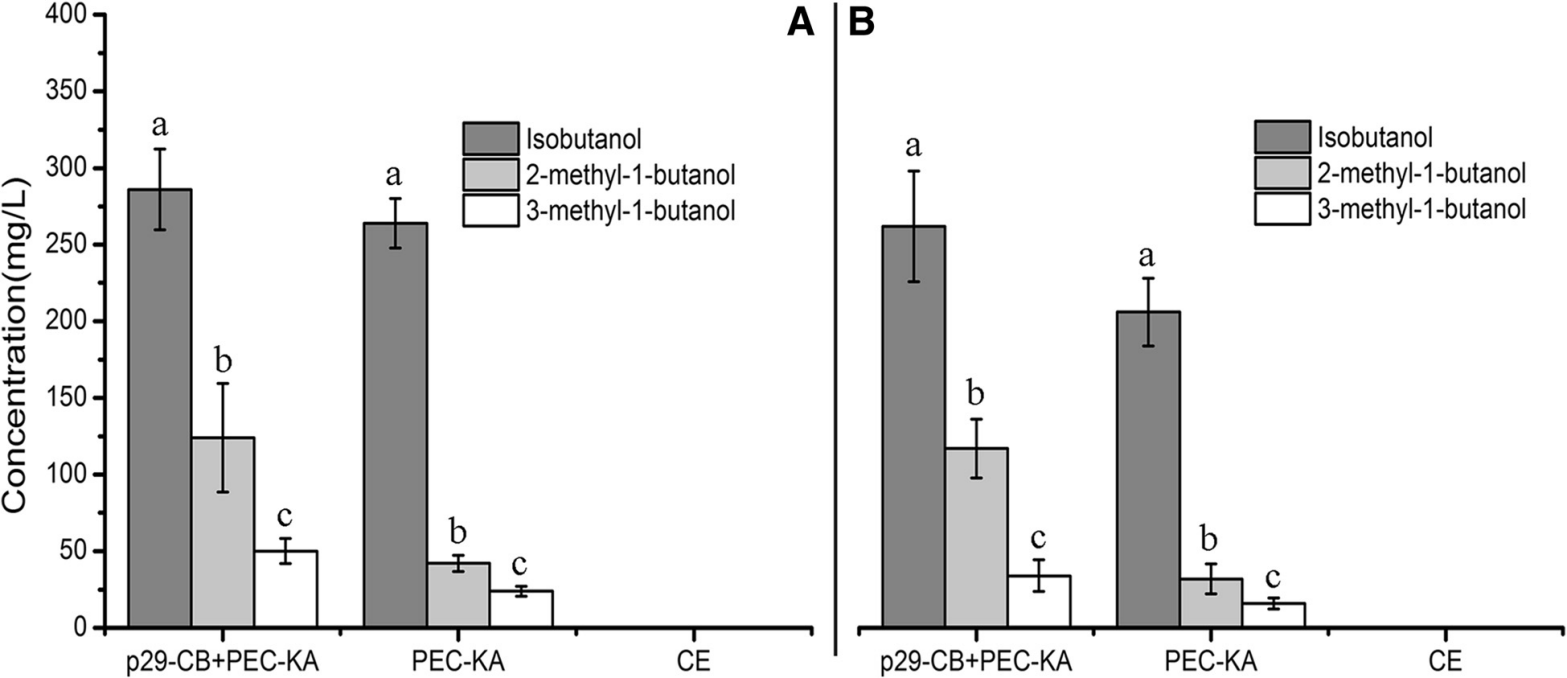
**Production of higher alcohols via construction of metabolic pathway C in the bioengineered strain of** ***C. crenatum*** **.** (6**A**) Yield of higher alcohols from glucose. (6**B**) Yield of higher alcohols from the acid hydrolysates of duckweed. p29-CB + PEC-KA: *BAT2* + *KIVD-ADH2*. CE: *C. crenatum* CICC 20153. Error bars indicate +/− SD (n = 3). The different letters indicate significant differences based on multiple comparisons for different alcohol products (*P* < 0.05).

Isobutanol yields were improved markedly by introduction of the gene *BAT2* (metabolic pathway C, p29-CB) into *C crenatum,* reaching 286.35 mg/L from glucose (Figure 6A) and 262.18 mg/L from duckweed (Figure 6B). Further, overexpressing the gene *BAT2* resulted in a higher relative proportion of 2-methyl-1-butanol and 3-methyl-1-butanol in the total yield versus 3-methyl-1-butanol (Figure 6). Overall though, there were greater impacts on isobutanol than on yields of the other two kinds of alcohol, so we infer that the gene *BAT2* plays an important role in the production of isobutanol when new metabolic pathways are constructed.

**Figure 7:**
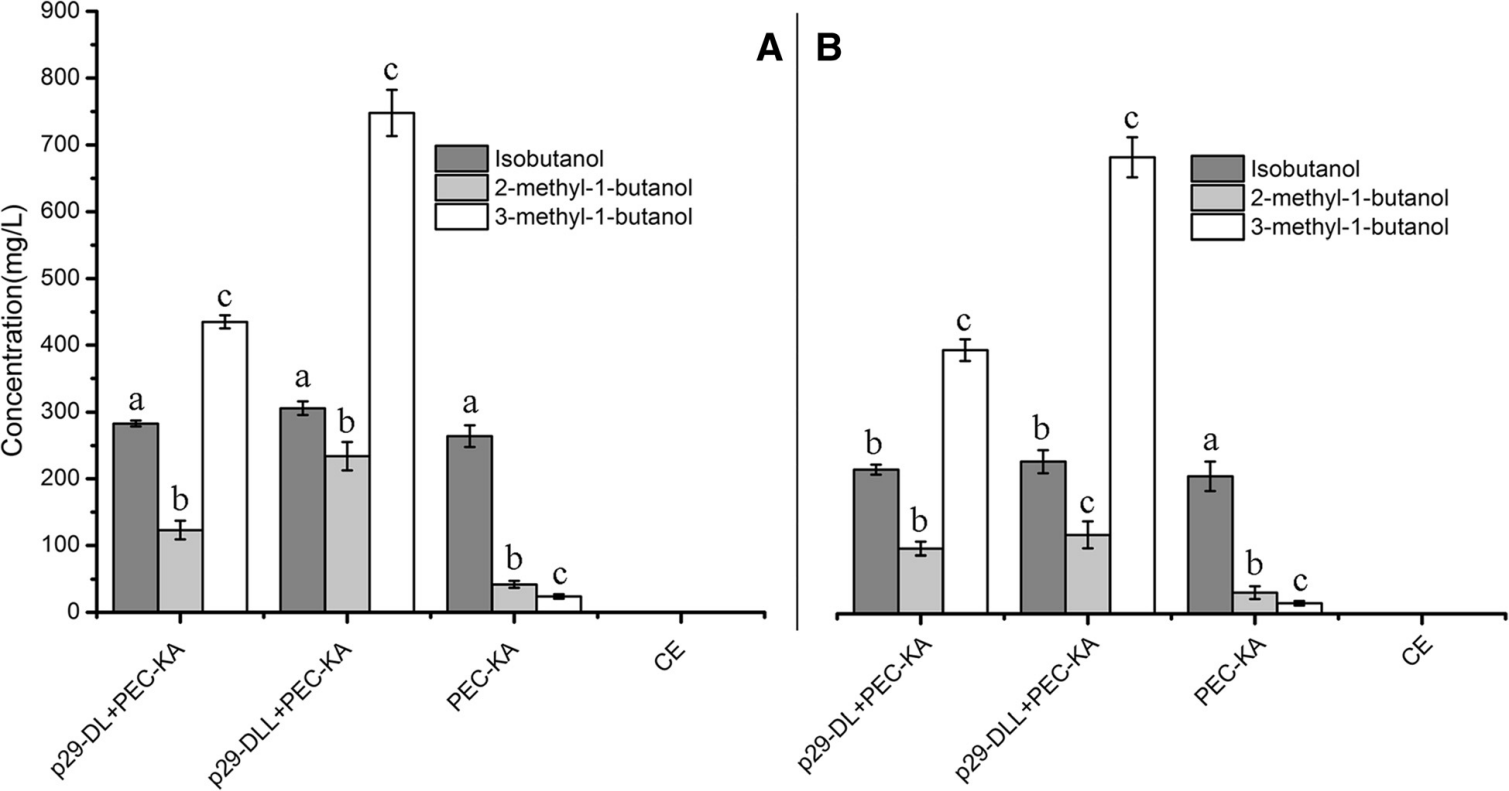
**Production of higher alcohols via construction of metabolic pathway D in the bioengineered strain of** ***C. crenatum*** **.** (7**A**) Yield of higher alcohols from glucose. (7**B**) Yield of higher alcohols from the acid hydrolysates of duckweed. p29-DL + PEC-KA: *LEU4* + *KIVD-ADH2*; p29-DLL + PEC-KA: *LEU4-LEU1* + *KIVD-ADH2*. CE: *C. crenatum* CICC 20153. Error bars indicate +/− SD (n = 3). The different letters indicate significant differences based on multiple comparisons for different alcohol products (*P* < 0.05).

**Figure 8:**
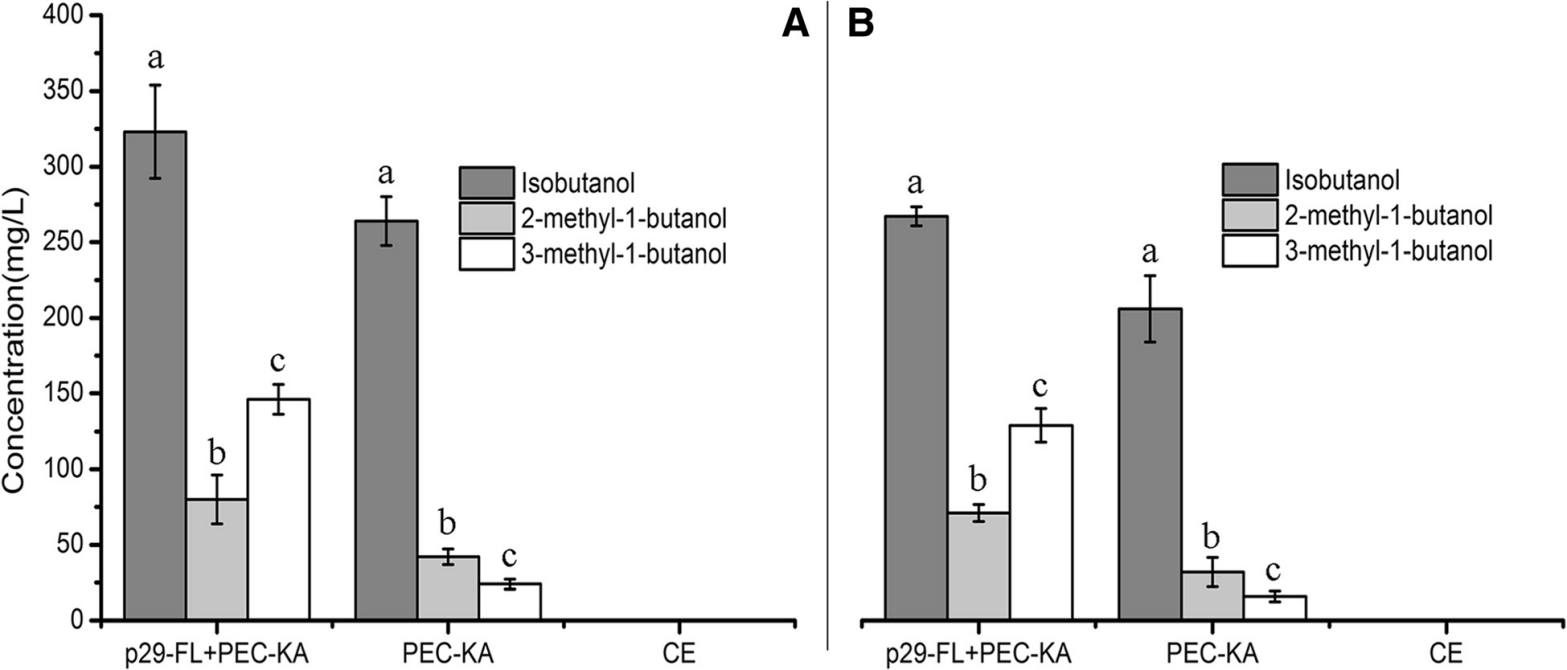
**Production of higher alcohols via construction of metabolic pathway E in the bioengineered strain of** ***C. crenatum*** **.** (8**A**) Yield of higher alcohols from glucose. (8**B**) Yield of higher alcohols from the acid hydrolysates of duckweed. p29-FL + PEC-KA: *LEU1* + *KIVD-ADH2*;. CE: *C. crenatum* CICC 20153. Error bars indicate +/− SD (n = 3). The different letters indicate significant differences based on multiple comparisons for different alcohol products (*P* < 0.05).

When metabolic pathway D was used, the highest yield of 3-methyl-1-butanol was obtained when the serial genes *LEU4-LEU1* (p29-DLL) were overexpressed into the host *C crenatum* (Figure 7). The maximum yield of 3-methyl-1-butanol was 748.35 mg/L from glucose substrates (Figure 7A, Table 4) and 684.79 mg/L from duckweed (Figure 7B). However, the yield dropped to 400 mg/L when only the gene *LEU4* (p29-DL) was overexpressed (Figure 7A). We also surveyed the production of other higher alcohols such as isobutanol and 2-methyl-1-butanol via this pathway; when only the gene *LEU4* was introduced, isobutanol and 2-methyl-1-butanol yields did not improve, compared to the use of only a vector (PEC-KA). The yield of 3-methyl-1-butanol improved significantly when the two genes *LEU4-LEU1* were introduced, but isobutanol and 2-methyl-1-butanol production did not change. With respect to metabolic pathway E, introducing only the gene *LEU1* (p29-FL) into *C. crenatum* yielded 146.47 mg/L of 3-methyl-1-butanol from glucose (Figure 8A) and 129.18 mg/L from duckweed (Figure 8B).

Overall, we showed that different yields of higher alcohols can be obtained by using yeast genes to construct new metabolic pathways in *C. crenatum*, and the bioengineered strain can successfully ferment duckweed hydrolysates to produce higher alcohols.

## Discussion

In this study, we engineered *C. crenatum* to induce the ability for higher alcohol production, and to further tap the potential capacity of this bioengineered strain for producing higher-chain alcohols such as isobutanol, 2-methyl-1-butanol and 3-methyl-1-butanol from the hydrolysates of duckweed. A key objective was to highlight the potential for using this host to construct higher alcohol production platforms. This is the first time we investigated the production of higher alcohols using *C. crenatum* as the expression host.

A body of research has demonstrated over recent years that major technical breakthroughs in metabolic engineering have enabled enormous progress in the production of higher alcohols, namely by building new metabolic pathways in bioengineered microbial hosts [43]. An important determinant of success is the identification of an appropriate host to express the relevant target genes. Although efforts to construct bioengineered strains of microorganisms such as *E. coli* and *C. glutamicum* to produce isobutanol have been successful—using the genes *alsS* from *Bacillus subtilis* and *ilvC* and *ilvD* from *E.coli* [10,44]—no attempts that we know of have been published using other enzymes to engineer corynebacterium. In order to obtain high yield of higher alcohols as much as possible, we try to develop relevant enzymes from other sources. In this paper, we made the first investigation to use yeast genes to build the metabolic pathways in *C. crenatum* for higher alcohol production. We constructed five metabolic pathways using genes from the isoleucine and leucine metabolic pathways of *S. cerevisiae* to produce higher alcohols. We investigated whether each pathway was capable of ultimately generating the desired end product, higher alcohols. The results showed that the bacterium *C. crenatum* can be used as an ideal expression host for producing alcohols using yeast genes, and that it has a strong ability to produce a wide range of long-chain alcohols.

Our findings demonstrate that the five metabolic pathways can prompt bioengineered strains to produce higher alcohols, with different pathways yielding the maximal production of the various desired products. For metabolic pathway A, the bioengineered strain with (p29-AL) + (PEC-KA) produced almost double the yield of 2-methyl-1-butanol compared to results with only PEC-KA, triple the yield of 2-methyl-1-butanol yield with (p29-ALI + PEC-KA) compared to (p29-AL) + (PEC-KA), and double the 2-methyl-1-butanol yield with (p29-ALII) + (PEC-KA) compared to (p29-ALI) + (PEC-KA)—that is, the tendency was the same, whether the substrate was glucose or duckweed (Figure 4A,B). For metabolic pathway B, the yield of isobutanol did not increase when two vectors were used (p29-BI) + (PEC-KA) compared to only PEC-KA, while production of isobutanol tripled from (p29-BI) + (PEC-KA) to (p29-BII) + (PEC–KA), and doubled from (p29-BII) + (PEC-KA) to (p29-BIII) + (PEC-KA), a conclusion confirmed by Figure 5(A,B). For metabolic pathway D, the change from using one (PEC–KA) to two vectors (p29-DL + PEC–KA) in the bioengineered strain tripled the yield of 3-methyl-1-butanol, but the change from (p29-DL) + (PEC–p29) to (p29-DLL) + (PEC–KA) caused the yield of 3-methyl-1-butanol to decline by 50% (Figure 7A,B). These results showed clearly that it is feasible to use *C. crenatum* as a host to bioengineer strains to produce higher alcohols, and also showed its potential as a bioengineered platform for producing biofuel.

As duckweed is a new kind of energy crop, some preliminary studies have been conducted using its hydrolysates to produce higher alcohols by microbial transformation [20]. Those results illustrated that duckweed has very high energy efficiency for producing biofuels that contain higher alcohols such as butanol and 2-methyl-1-butanol, identifying its great potential for use as an industrial feedstock in the production of biofuels as alternative energy. Further research and development should concentrate on the production of higher alcohols via fermentation of duckweed by appropriate microorganisms. Our research provides a foundation for the development of industrialized biofuel production using duckweed. However, the yields of higher alcohols from duckweed are extremely low (especially C_5_ alcohols such as 2-methyl-1-butanol and 3-methyl-1-butanol), making it necessary to develop an optimal host as a cell factory to match the fermentation substrate of duckweed, in order to obtain the highest possible yields. In this paper, in order to rudimentarily evaluate the ability of this engineered host for fermenting duckweed to produce alcohols, we chose preferentially acid hydrolysates of duckweed as fermentation substrate, rather than eznymatic hydrolysates. We suppose if this engineered strains can ferment acid hydrolysate with inhibitors, then it can certainly ferment enzymatic hydrolysate without inhibitors, because formation of inhibitors from acid hydrolysis would likely inhibit the gowth of bacterium, lead to inefficiency of fermentation. Our results confirmed that it is feasible to bioengineer *C. crenatum,* via the construction of new metabolic pathways with yeast genes, to ferment acid hydrolysates of duckweed to produce higher alcohols. Each individual metabolic pathway that we tested generated similar results using glucose or duckweed. Notably, the desired results were achieved only when multiple exogenous genes were overexpressed at the same time.

Each metabolic pathway led to the production of higher alcohols by its corresponding bioengineered strain, and seemingly generated greater yields of higher alcohols using glucose as the fermentation substrate versus duckweed. However, statistical comparison of yields from the two different fermentation substrates, on the whole showed that the yield of each kind of higher alcohol from duckweed was not significantly lower than the yield from glucose (Figure 4~Figure 8, *P* <0.05). For example, there were almost no differences for 2-methyl-1-butanol (Figure 4A, B). This showed that the presence of some anti-nutritive compounds in the acid hydrolysates of duckweed did not significantly inhibit the growth of bioengineered *C. crenatum*, enabling nearly equal yields compared to those from glucose under the same fermentation conditions. This conclusion was confirmed when we tested for the presence of inhibitors from acid hydrolysate of duckweed, including furfural, hydroxymethylfurfural (HMF) and other phenols reported to affect fermentation [45]. In this experiments, only furfural and HMF were detected (Table 5). Comparison of fermentation results via two substrates confirmed the low levels of inhibitors detected in the pretreated substrate (furfural and HMF) do not significantly inhibit bacterial growth or production, also showed inhibitors concentrations were below levels to disrupt the growth of *C. crenatum*.

**Table 5:** **Inhibitor determination from acid hydrolysates of duckweed**

**Compound**	**Inhibitor concentration (mg/L)**
Furfural	19.65 ± 0.81
Hydroxymethylfurfural (HMF)	3.89 ± 0.25
Glucuronic acid	ND
*p*-coumaric acid	ND
Syringic acid	ND
Ferulic acid	ND

ND: Not detected.

Overall, our analyses demonstrate that duckweed has potential as a biofuel crop, even more so than plants currently used for cost-saving, environment-friendly purpose, such as corn or cassava. In fact, duckweed would be even more promising for producing higher alcohols if further developments can be achieved by constructing even more effective bioengineered hosts.

The combined results of this study suggest that although the genetic background of *C. crenatum* is not clear, its genome-scale metabolic network is similar to that of *C. glutamicum* because it is a homologous strain. Thus, the genetic tools used for metabolic engineering of *C. glutamicum*, and experience gained in the development and utilization of that species*,* can inform the production of higher alcohols using *C. crenatum* as a host platform. Lessons can also be drawn from the metabolic pathways of carbon flow in *C. glutamicum* to produce higher alcohols.

## Conclusions

In summary, it is clearly feasible to use *C. crenatum* as a host to bioengineer strains with efficacious abilities for producing higher alcohols. We report here the first attempt to use *C. crenatum* for this purpose. Our experiments showed that effectively expressing metabolic enzymes in combinations that are associated with higher alcohol production in the metabolic pathways of *S. cerevisiae* led to accumulation of the precursor of amino acids, which enabled genetic engineering of the strain to produce higher alcohols. Our experimental results will facilitate further development of this host to produce higher alcohols. Our results also showed that duckweed plants represent an ideal fermentation substrate for the development of higher alcohols. This provides a solid foundation for the use of duckweed as the next generation of alternative energy feedstocks.
